# The Patient Typology about deprescribing and medication-related decisions: A quantitative exploration

**DOI:** 10.1111/bcpt.13911

**Published:** 2023-07-03

**Authors:** Kristie Rebecca Weir, Aaron M. Scherer, Sarah E. Vordenberg, Sven Streit, Jesse Jansen, Katharina Tabea Jungo

**Affiliations:** 1 Institute of Primary Health Care (BIHAM), University of Bern, Bern, Switzerland; 2 Sydney School of Public Health, Faculty of Medicine and Health, The University of Sydney, Sydney, Australia; 3 University of Iowa Carver College of Medicine, Iowa City, Iowa, USA; 4 Department of Clinical Pharmacy, University of Michigan College of Pharmacy, Ann Arbor, Michigan, USA; 5 Faculty of Health, Medicine and Life Sciences, School for Public Health and Primary Care, Maastricht University, Maastricht, Netherlands

**Keywords:** attitudes towards medicines, communication, consumer preferences, quantitative research methods polypharmacy, shared decision-making

## Abstract

This study aimed to test the adequacy of a quantitative measure of our qualitatively developed Patient Typology—categories of older adults’ attitudes towards medicines and medicine decision-making—and identify characteristics associated with each Typology. We conducted secondary data analyses of a subset of survey item measures of adults (≥65 years) who were members of online survey panels in Australia, the United Kingdom, the United States and the Netherlands (*n* = 4688). Multinomial logistic regression analyses assessed associations between demographic, psychosocial and medication-related measures. Mean age was 71.5 (5), and 47.5% of participants were female. Factors associated with an increased likelihood of identifying with Typology 1 ‘Attached to medicines’ over Typology 2 ‘Open to deprescribing’ were higher positive attitude towards polypharmacy (RRR = 1.12, *p* = <0.001) and higher need for certainty (RRR = 1.11, *p* = 0.039). Factors associated with an increased likelihood of identifying with Typology 3 ‘Defers (medication decision-making) to others’ over Typology 2 were older age (RRR = 1.47 per 10-year age increase, *p* = <0.001) and a decreased likelihood of prior deprescribing experience (RRR = 0.73, *p* = 0.033). This study provides validation of the Typology with large samples from four countries, with the quantitatively-measured typologies generally aligning with the qualitatively derived categories. Our Patient Typology measure provides a succinct way researchers can assess attitudes towards deprescribing.

## 1 | INTRODUCTION

Internationally, there is increasing focus on the harms of prolonged medication use in the older population. Recent international data indicate that 39%–45% of older adults engage in polypharmacy: taking five or more medications daily.^1–4^ A medication is considered *inappropriate* when potential harms of continuing the medication outweigh its potential benefits for an individual.^5^ A medication could also be considered inappropriate when it does not align with an individual’s goals and preferences.^6^ One way to reduce medication-related harm is by deprescribing through dose reduction or discontinuing selected medicines.^6^ However, deprescribing can be a challenging process, and consideration of the clinician and patient attitudes towards medicines is necessary for collaborative deprescribing.

Attitudes towards medicines and openness to deprescribing influence how willing older persons are to make changes to their medicines. Older adults can feel reluctant to deprescribe and may have unrealistic beliefs about the benefits of their medications.^7,8^ If older adults have been told they probably need to take a medication for the rest of their life, discussion of possible deprescribing may make them anxious or sceptical.^9^ Older adults may presume their medication is of high importance if they have been taking medication for many years.^10^ Further to this, older adults’ preferences may change over time,^11^ and deprescribing decisions can be influenced by specific medications or with a change of the patients’ health status.^12,13^

Even if aspects of the deprescribing recommendation might make people more or less likely to deprescribe, people may differ in their baseline attitudes towards medicines. Researchers have used typologies to make sense of patterns and to categorize differences in how older adults perceive their medications. Previous typologies have categorized participants in relation to deprescribing cardiovascular^14^ and cardiometabolic medication,^15^ self-management of medications^16^ and decision-making preferences.^17,18^ However, none of these typologies focused on deprescribing non-specific medications.

**FIGURE 1:**
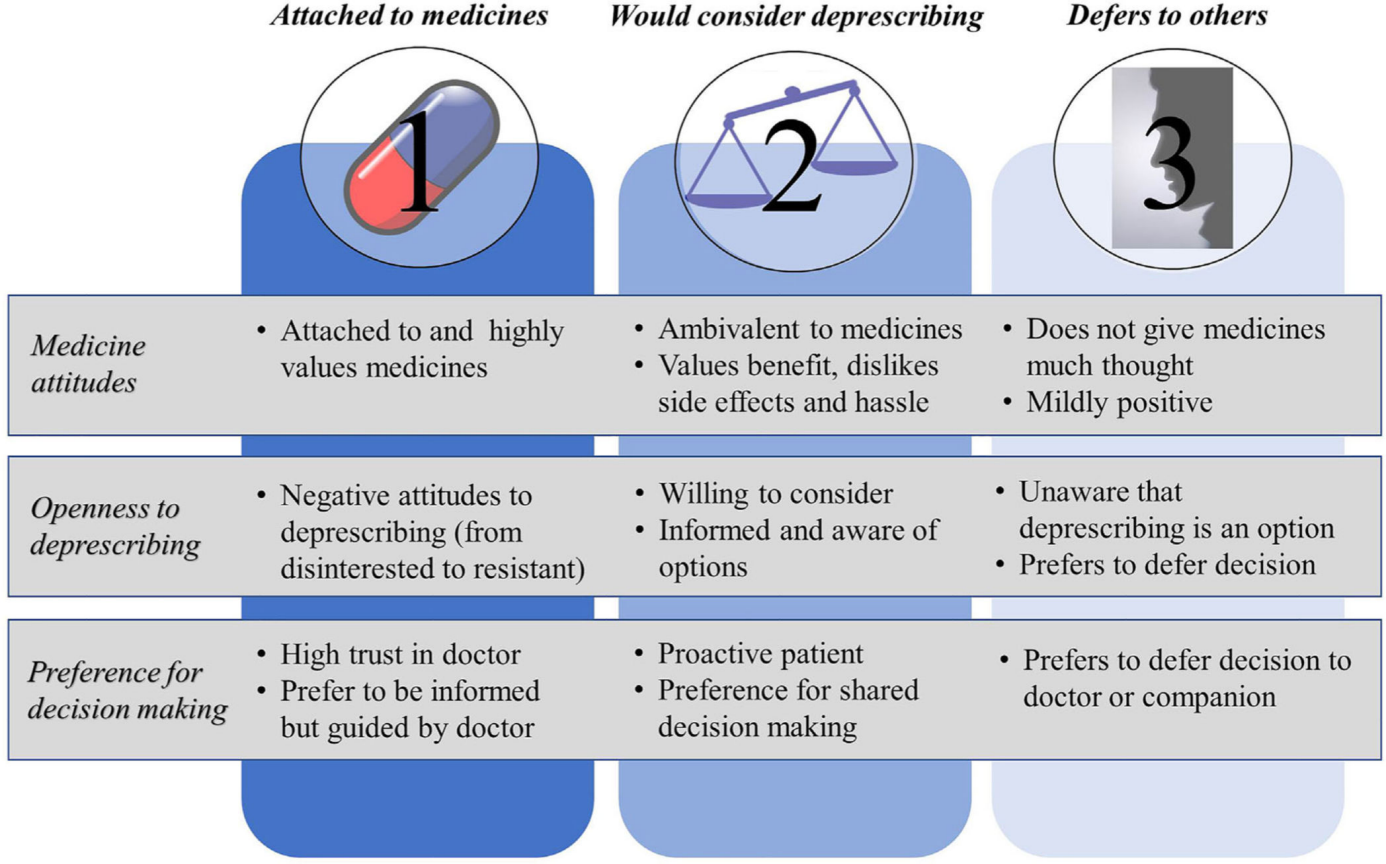
Patient Typology (qualitatively-developed) Legend: Figure from Weir et al., 2018

Our previous qualitative work has explored the nuances of older adults’ deprescribing and decision-making preferences, where positive and negative attitudes towards medicines can often coincide.^8^ This has led to the development of the Patient Typology,^8^ which categorizes three typologies of patients in terms of their attitudes towards medicines, willingness to deprescribe and their decision-making styles (‘Attached to medicines’, ‘Would consider deprescribing’ and ‘Defers (medication decision-making) to others’). Our Patient Typology (Figure 1) has been rigorously developed using qualitative methods and informed by a theoretical shared decision-making framework.^19^ It is gaining interest in the deprescribing field and has been used in interventional studies,^20,21^ a qualitative study^15^ and survey study.^22^ This indicates that it is applicable in a variety of deprescribing studies. However, qualitative methods can be time consuming. Thus, a short, quantitative Patient Typology measure would be easier to incorporate into research and practice. Our study aims were to develop a quantitative Patient Typology measure and identify demographic, psychosocial and medication-related characteristics associated with each Typology category.

## 2 | METHODS

### 2.1 | Development of the quantitative measure of the Patient Typology

We used an iterative process to develop a quantitative Patient Typology measure that included descriptions that summarized the key aspects of the three Patient Typology categories.^8^ Input was received from 10 multi-disciplinary researchers and clinicians, which included experts in geriatrics, general practice, pharmacy, health literacy, ethics, health psychology and shared decision-making and a consumer representative. Feedback was provided in one-to-one in-person discussions, in meetings, over the phone or via email. This was followed by informal pilot testing in Australia with seven older adults and two caregivers of older adults (in person and over the phone), resulting in minor wording changes so the descriptions were easier to understand.

### 2.2 | Study design and sample

This study was part of a larger survey-based online experiment testing medication-related factors that influence older adults’ preferences for deprescribing.^23^ Participants were asked to reflect on a scenario in which a general practitioner recommended stopping either (1) simvastatin for the prevention of heart disease and stroke or (2) lansoprazole for the treatment of indigestion. The survey was completed by older adults aged 65 years and above, recruited from Australia, the United Kingdom, the United States and the Netherlands. Participants were recruited through a panel of Internet users administered by Qualtrics Research Panels (Provo, UT) from December 2020 through March 2021. Opt-in methods, sample requirements and sample quotas were utilized to ensure the samples were demographically diverse and eligible panellists were randomly invited. Full details of the study have been reported elsewhere.^23^ This study received exempt status approval from the University of Michigan Institutional Review Board (IRB). The original study was conducted in accordance with the *Basic & Clinical Pharmacology & Toxicology* policy for experimental and clinical studies and was registered with ClinicalTrials.gov, NCT04676282.^24^

### 2.3 | Survey

The self-assessed survey was administered in English for participants in the United Kingdom, the United States and Australia and in Dutch for participants in the Netherlands. The survey was translated from English to Dutch by one of the co-authors (JJ) and a medical student from the Netherlands. Minor changes to wording were made when necessary to fit with the context of each country (e.g. primary care provider vs. general practitioner).

### 2.4 | Primary outcome measure

The primary outcome for this study was which of the three Patient Typology descriptions^8^ participants most closely identified with. The order in which the three descriptions were presented was randomized to prevent order bias. See Supporting Information for the descriptions.

### 2.5 | Patient characteristics

The demographic, psychosocial and medication-related variables included were based on hypothesized relationships with the Patient Typology, a systematic review and meta-analysis of peoples’ attitudes towards deprescribing,^25^ and prior deprescribing research about barriers and facilitators, communication and shared decision-making by the co-authors.^7,19^ The variables included in the analysis were attitudes towards medications and deprescribing, personality traits and health preferences, health characteristics, demographics and medication use.

Attitudes towards medications and deprescribing:

- *Agreement with deprescribing recommendation (for simvastatin or lansoprazole) from a general practitioner*: Participant agreement with a hypothetical deprescribing recommendation on a 6-point Likert scale, with *strongly disagree* (1) and *strongly agree* (6) as the scale anchors^23^
- *Perception of harmfulness of deprescribing*: Perceived potential harm of deprescribing on a 10-point Likert scale, with *not harmful* (1) to *very harmful* (10) as the scale anchors^26,27^
- *Beliefs about Medicines Questionnaire (BMQ) Harm and Overuse subscales (eight items)*: Beliefs about medicines in general focusing on harmfulness and overuse on a 5-point Likert scale, with *strongly disagree* (1) to *strongly agree* (5) as the scale anchors^28,29^
- *Attitudes towards polypharmacy*: Attitude towards taking 11 medications on a 10-point Likert scale, with *very negative* (1) and *very positive* (10) as the scale anchors^30^

Personality traits and health preferences:

- *Medical Maximizer-Minimizer (MM1)*: Preferences for seeking more or less medical care, ranging from ‘I strongly lean towards waiting and seeing (1)’ to ‘I strongly lean towards taking action (6)’^31^
- *Need for certainty scale*: Comfort or discomfort with uncertainty on a 5-point Likert scale, with *strongly disagree* (1) and *strongly agree* (5) as the scale anchors^32^
- *Health Regulatory Focus Scale (HRFS) Health Promotion subscale (six items)*: Preference for engaging in actions to promote health on a 7-point Likert scale, with *not at all* (1) to *to a great extent* (7) as the scale anchors^33^

Health characteristics:

- *Self-rated general health*: General health rating on a 5-point Likert scale, with *poor* (1) to *excellent* (5) as the scale anchors^34^
- *Health literacy*: Confidence in filling out medical forms on a 5-point Likert scale, with *not at all* (1) to *extremely* (5) as the scale anchors^35,36^

Mean values were calculated for the following variables: BMQ General, HRFS Health Promotion and the Need for Certainty scale. Due to high collinearity, we did not include the BMQ Specific or the HRFS Health Prevention subscales. See Supporting Information for variables included in the current analyses and specific item wording.

Demographics and medication use:

Demographics included age, gender, education, relationship status and living situation. Medication use measured included self-reported number of medications (prescription, non-prescription and/or dietary supplements), level of support for managing their medications and prior experience taking a medication from the same therapeutic class as the medication in the scenario (HMG-CoA reductase inhibitor or proton pump inhibitor).

### 2.6 | Statistical analysis

We calculated descriptive statistics for each typology. Categorical variables are presented as frequencies and percentages; means and the standard deviations are presented for scales and continuous measures. We used multilevel multinomial logistic regression analyses accounting for the clustering effect at country level to calculate the relative risk of choosing a certain Patient Typology (Supporting Information). Typology 2 ‘Would consider deprescribing’ was used as the base outcome in these analyses as it was the most selected typology. Demographic characteristics (age, gender, education, health literacy, health status), risk attitudes, personality traits and medication-related characteristics (number of medications used, personal use of the medication presented in the scenario) were included in the models as predictor variables. Subgroup analyses revealed no major differences between the two medication types (simvastatin and lansoprazole), so we chose to report results collapsed across the two medication types for simplicity. All analyses were conducted with Stata, version Stata SE 16.0 (StataCorp).

## 3 | RESULTS

### 3.1 | Participant characteristics

In total, 5693 individuals started the survey, and 5311 completed it.^23^ We excluded 301 participants who were ineligible for participation (less than 65 years or did not reside in a participating country) and 81 participants who did not agree to give high-quality answers. We excluded 623 participants who had not responded to the Typology question (Table S1).

Participant characteristics are presented in Table 1. In total, 4688 participants (88% of the final sample) completed the Patient Typology question. In each of the four countries, Typology 2 ‘Would consider deprescribing’ was the most selected typology, and Typology 3 ‘Defers (medication decision-making) to others’ was the least selected typology.

### 3.2 | Multinomial logistic regression analysis

The results from the multinomial logistic regression analysis are shown in Table 2 and summarized in Box 1. The multinomial logistic regression shows the likelihood of a participant choosing ‘Attached to medicines’ Typology 1 or ‘Defers (medication decision-making) to others’ Typology 3 over ‘Would consider deprescribing’ Typology 2, which has been defined as the base outcome. Variables that were significantly associated with the likelihood of selecting a specific typology over the reference category are summarized in Table S2.

#### 3.2.1 | Selecting Typology 1 ‘Attached to medicines’ over Typology 2 ‘Would consider deprescribing’

Factors associated with an increased likelihood of primarily identifying with Typology 1 ‘Attached to medicines’ (vs. Typology 2 ‘Would consider deprescribing’) were older age (RRR = 1.26 per 10-year increase in age, *p* < 0.01), higher positive attitude towards polypharmacy (RRR = 1.12, *p* = <0.001) a higher need for certainty (RRR = 1.11, *p* = 0.039) and perceiving deprescribing as potentially more harmful (RRR = 1.04, *p* = 0.047) (Table 2).

Factors associated with a reduced likelihood of primarily identifying with ‘Attached to medicines’ Typology (vs. ‘Would consider deprescribing’ Typology) were being female (RRR = 0.78, *p* < 0.01), higher education level including trade school/college or associate’s degree (RRR = 0.78, *p* < 0.01), bachelor’s degree (RRR = 0.58, *p* < 0.001) or Master’s degree (RRR = 0.76, *p* = 0.04), higher confidence filling out medical forms (‘somewhat’ RRR = 0.49, *p* = 0.04; ‘quite a bit’ RRR = 0.39, *p* < 0.01; ‘extremely’ RRR = 0.35, *p* < 0.01) belief that medicines were over-used or harmful (RRR = 0.47, *p* < 0.001) and previous experience with deprescribing (RRR = 0.73, *p* < 0.01).

Results from the multinomial logistic regression analyses in relation to the hypotheses are summarized in Table S2. Consistent with the hypotheses, participants who selected ‘Attached to medicines’ Typology (vs. ‘Would consider deprescribing’ Typology) were more likely to: perceive deprescribing as harmful, have less experience with deprescribing, more positive attitudes towards polypharmacy, lower beliefs that medicines are over-used or harmful, lower health literacy, lower education level, and a higher need for certainty. Contrary to the hypotheses, less agreement with deprescribing was not confirmed to increase/decrease the likelihood of choosing ‘*Attached to medicines*’ *Typology over* ‘*Would consider deprescribing*’ *Typology*.

#### 3.2.2 | Selecting Typology 3 ‘Defers (medication decision-making) to others’ over Typology 2 ‘Would consider deprescribing’

Factors associated with an increased likelihood of primarily identifying with Typology 3 ‘Defers (medication decision-making) to others’ (vs. Typology 2 ‘Would consider deprescribing’) were older age (RRR = 1.47 per 10-year increase in age, *p* < 0.001), higher agreement with deprescribing (RRR = 1.09, *p* = 0.034), and general health reported as excellent (RRR = 3.04, *p* < 0.001).

Factors associated with a reduced likelihood of primarily identifying with ‘Defers (medication decision-making) to others’ Typology (vs. ‘Would consider deprescribing’ Typology) were being female (RRR = 0.48, *p* < 0.001), higher education level including trade school/college or associate’s degree (RRR = 0.59, *p* < 0.001), bachelor’s degree (RRR = 0.41, *p* < 0.001) or master’s degree (RRR = 0.43, *p* < 0.001), confidence filling out medical forms (‘quite a bit’ RRR = 0.41, *p* = 0.02; ‘extremely’ RRR = 0.28, *p* < 0.01), leaned towards taking action in relation to their health (RRR = 0.88, *p* < 0.01), greater desire of participants to engage in actions to promote good health (RRR = 0.80, *p* < 0.001) and experience with deprescribing (RRR = 0.73, *p* = 0.03) (Table 2).

For ‘Defers (medication decision-making) to others’ Typology, the multinomial logistic regression results that were consistent with the hypotheses (Table S2) were older age, male, slightly higher agreement with the deprescribing recommendation, less experience with deprescribing, lower health literacy, lower education level, less desire to engage in actions to promote their health and a preference towards waiting and seeing. Contrary to the hypotheses, excellent health increased the likelihood of choosing ‘Defers (medication decision-making) to others’ Typology over ‘Would consider deprescribing’ Typology. Also, lower beliefs that medicines are over-used or harmful or less need for certainty were not confirmed to increase/decrease the likelihood of choosing ‘Defers (medication decision-making) to others’ Typology over ‘Would consider deprescribing’ Typology.

## 4 | DISCUSSION

In the current study, we tested for changes in the relative risk of identifying with one of the three Patient Typologies for demographic, psychosocial and medication-related variables theorized to be associated with each Typology. Across the three Typologies, the quantitative results were generally consistent with the hypotheses.

To our knowledge, this is the first validation of a qualitative-derived deprescribing and medication decision-making typology using quantitative methods in a large sample of older adults from multiple countries. Our findings were consistent with the hypotheses for most measures within these categories: deprescribing, attitudes towards medicines, knowledge about medicines and health, decision-making preferences and characteristics. We conclude that our quantitative measure can be used to assess the Patient Typology and the few discrepancies found, which will be discussed in detail, do not undermine its validity.

For ‘Attached to medicines’ Typology participants, we would expect to see resistance to deprescribing. Although participants who selected ‘Attached to medicines’ over ‘Would consider deprescribing’ Typology perceived deprescribing to be more harmful and had less experience with deprescribing—consistent with the hypotheses—less agreement with a deprescribing recommendation was not significant. This may be due to a ceiling effect—meaning there was not enough variability to detect differences, as the majority of participants in the larger study agreed with the deprescribing recommendation (>80%).^23^

With ‘Defers (medication decision-making) to others’ Typology 3, participants who reported their general health as excellent had an increased likelihood of more than three times of identifying with this Typology over the ‘Would consider deprescribing’ Typology. From the hypotheses, we would expect participants identifying with ‘Defers (medication decision-making) to others’ Typology to report their health as fair or poor. A consideration is that few participants overall (approximately 5% or lower) reported their health level as poor, which was seen across all participants and typologies. Also, agreement with a deprescribing recommendation was associated with an increased likelihood of identifying with ‘Defers (medication decision-making) to others’ Typology over ‘Would consider deprescribing’ Typology. Given that participants had less experience with deprescribing, this may reflect this group’s agreement with a general practitioner’s recommendation rather than deprescribing itself—which would be consistent with our qualitative work.

There is utility in using the Patient Typology as a target for deprescribing intervention development. It is important that interventions consider the complex nature and interaction of older adults’ attitudes, beliefs and decision-making in deprescribing. Current deprescribing interventional research frequently relies on the notion that older adults want to reduce or stop their medications. However, this does not align with real-life clinical practice, where clinicians find it difficult to stop medications due to patient preferences and older adults commonly prefer to continue them.^37^ This is reflected in deprescribing studies where up to 75% of older adults decline to participate,^38–40^ and the challenges of implementing deprescribing are well known.^41^ Additional work is needed to develop a validated measure that uses a more nuanced approach to categorize older adults who are more attached to their medications and may be concerned about the potential harms of deprescribing.

A strength of our work is that it examined the typologies in a sample of older adults across four countries with different healthcare systems. This important work offers further confirmation of the Patient Typology, giving insight into whether participants can self-select their typology and provides evidence for using these questions to assess the typologies in deprescribing research. Exploring the Typologies quantitatively is a useful way to gain further understanding and to develop more practical ways to apply the measure in ‘real life’. Another strength of our study is that we included commonly used, validated scales. Although deprescribing decisions are often influenced by medication type, our findings were similar for both medication scenarios (simvastatin and lansoprazole). This suggests the Typology may be applicable for different medications and deprescribing decisions. However, contextual factors that influence people to be more or less likely to deprescribe does not mean that people cannot systematically differ in their baseline attitudes towards medicines and deprescribing.

A limitation of this study is that participants were well enough to participate in an online survey and we may have recruited less older adults who vary in their health status, function and frailty level. For example, most participants in this study reported relatively high levels of self-rated health given the age of the population and were quite a bit or extremely confident filling out medical forms. The variables included in our analysis were determined by the data collected as part of a larger study. Therefore, we may be missing important variables such as older adults’ trust in their doctor and decision-making preferences, which will be examined in our future work. Also, there remains a need to explore the relationship between the typologies and actual deprescribing as the design of our study did not allow for that. Although the findings were generally in line with the hypotheses, it is understandable that a single quantitative measure may not perfectly capture the nuances of an in-depth interview. Therefore, our future work will split these items so that participants are able to select their own combinations of attitudes towards medicines, knowledge, deprescribing and decision-making preferences to identify other typologies that may exist.

We have explored a typology of older people with regards to deprescribing using quantitative methods. Participants selecting Typologies 1, 2 and 3 differed in terms of their attitudes towards medicines, deprescribing preferences and demographic characteristics. Understanding the differences and commonalities of older adults in the context of medication-use is important. Utilizing the Patient Typology could be helpful to guide more effective decision-making and management of medicines in the older population. With this information, clinicians could target their communication to focus on the preferences of the older adult and perhaps streamline discussions about deprescribing. A measure such as this would not substitute a patient-GP relationship rather it may support tailored communication by bringing patient preferences to the foreground. Additionally, knowing which typology an individual identifies with could be useful for older adults themselves, by encouraging self-reflection, and may empower them in other health care interactions.

## Supplementary Material

Supplementary Tables

Study Data

## Figures and Tables

**TABLE 1:** Baseline characteristics.

	Total	Typology 1	Typology 2	Typology 3
	(*n* = 4688)	(*n* = 1446)	(*n* = 2464)	(*n* = 778)
	*n* (%)	*n* (%)	*n* (%)	*n* (%)
Country				
Australia	1098 (23.4)	371 (25.7)	554 (22.5)	173 (22.2)
Netherlands	1021 (21.8)	267 (18.5)	566 (23.0)	188 (24.2)
United Kingdom	1231 (26.3)	345 (23.9)	630 (25.6)	256 (33.0)
United States	1338 (28.5)	463 (32.0)	714 (29.0)	161 (20.7)
Gender				
Female	2226 (47.5)	631 (43.6)	1311 (53.2)	284 (36.5)
Male	2450 (52.3)	811 (56.1)	1147 (46.6)	492 (63.2)
Missing	12 (0.3)	4 (0.3)	6 (0.2)	2 (0.2)
Education				
High school diploma or less	1425 (30.4)	478 (33.1)	644 (26.1)	303 (39.0)
Trade school/some college/associate’s degree	1671 (35.6)	522 (36.1)	885 (35.9)	264 (33.9)
Bachelor’s degree	1057 (22.6)	280 (19.4)	633 (25.7)	144 (18.5)
Master’s degree or higher	532 (11.4)	165 (11.4)	300 (12.2)	67 (8.6)
Missing	3 (0.1)	1 (0.1)	2 (0.1)	0 (0)
Marital status				
Partnered/married	3073 (65.6)	949 (65.6)	1597 (64.8)	527 (67.7)
Not partnered/married	1613 (34.4)	497 (34.4)	865 (35.1)	251 (32.3)
Missing	2 (0.5)	0 (0)	2 (0.1)	0 (0)
Living situation				
Alone	1289 (27.5)	403 (27.9)	682 (27.7)	204 (26.2)
With someone	3221 (68.7)	974 (67.4)	1695 (68.8)	552 (71.0)
Nursing home or retirement village	21 (0.5)	7 (0.5)	11 (0.5)	3 (0.4)
Missing	157 (3.4)	62 (4.3)	76 (3.1)	19 (2.4)
Health literacy				
None	82 (1.8)	38 (2.6)	25 (1.0)	19 (2.4)
A little bit	145 (3.1)	43 (3.0)	62 (2.5)	40 (5.1)
Somewhat (potential for lower health literacy)	453 (9.7)	151 (10.4)	197 (8.0)	105 (13.5)
Quite a bit	1846 (39.4)	523 (36.2)	973 (39.5)	350 (45.0)
Extremely	2160 (46.1)	691 (47.8)	1206 (48.9)	263 (33.8)
Missing	2 (0)	0 (0)	1 (0)	1 (0.1)
Support for managing medications				
None	4080 (87.0)	1241 (85.8)	2174 (88.2)	665 (85.5)
Occasional support	373 (8.0)	118 (8.2)	195 (7.9)	60 (7.7)
Complete assistance	169 (3.6)	72 (5.0)	63 (2.6)	34 (4.4)
Missing	66 (1.4)	15 (1.0)	32 (1.3)	19 (2.4)
Self-reported health				
Poor	220 (4.7)	76 (5.3)	109 (4.4)	35 (4.5)
Fair	1209 (25.8)	420 (29.1)	617 (25.0)	172 (22.1)
Good	2041 (43.5)	605 (41.8)	1086 (44.1)	350 (45.0)
Very good	1023 (21.8)	292 (20.2)	555 (22.5)	176 (22.6)
Excellent	195 (4.2)	53 (3.7)	97 (3.9)	45 (5.8)
Missing	195 (4.16)	0 (0)	0 (0)	0 (0)
Previous experience with deprescribing (vs. none)	538 (11.5)	131 (9.1)	338 (13.7)	69 (8.9)
Missing	3 (0.1)	2 (0.1)	0 (0)	0 (0)
	Mean (SD)	Mean (SD)	Mean (SD)	Mean (SD)
Age	71 (4.9)	72 (5.2)	71 (4.6)	72 (5.3)
# of prescribed medications	4.9 (8.7)	5.7 (8.4)	4.9 (8.7)	3.7 (9.1)
# of over-the-counter medications/supplements	2.1 (4.3)	2.1 (4.8)	2.3 (4.2)	1.6 (3.2)
Medical Maximizing-Minimizing Preferences^a^ *(range: 1*–*6)*	3.4 (1.4)	3.7 (1.4)	3.4 (1.3)	3.1 (1.3)
Beliefs about Medicines Questionnaire General^b^ *(α* = *0.85, range: 1*–*5)*	2.6 (0.8)	2.3 (0.7)	2.7 (0.8)	2.8 (0.8)
Agreement with hypothetical deprescribing recommendation *(range: 1*–*6)*	4.8 (1.4)	4.8 (1.4)	4.8 (1.3)	4.9 (1.2)
Polypharmacy attitudes^c^ *(range: 1*–*10)*	4.0 (2.2)	4.7 (2.3)	3.7 (2.0)	3.5 (2.1)
Perceived harmfulness of deprescribing *(range: 1*–*10)*	4.0 (2.4)	4.2 (2.6)	3.9 (2.3)	3.8 (2.1)
Need for Certainty scale *(α* = *0.85, range: 1*–*5)*	3.6 (0.8)	3.7 (0.8)	3.6 (0.8)	3.4 (0.9)
Health Promotion scale^d^ *(α* = *0.87, range: 1*–*7)*	5.1 (1.1)	5.1 (1.1)	5.2 (1.1)	4.9 (1.2)

a Higher values indicating a stronger preference towards medical interventions.

b Higher values indicating a stronger belief that medicines are over-used or harmful.

c Higher values indicating more positive attitudes.

d Higher values indicating a stronger preference for engaging in actions to promote health.

**TABLE 2:** Multinomial logistic regression of the associations between patient characteristics and the three typologies^a^ (n = 4153).

Typology 2 Base outcome of the model	Typology 1			Typology 3		
***Typology 2***^a^ ‘Would consider deprescribing’: Ambivalent attitudes towards medicines, preferred a proactive role in decision-making, were open to deprescribing. Knowledgeable about their health or medications, accessed information. Reported very good or higher self-rated health	***Typology 1***^a^ ‘Attached to medicines’: Positive attitudes towards medicines, left decisions to their doctor, resistant to deprescribing. Some knowledge about their health or medications. Reported good self-rated health			***Typology 3***^a^ ‘Defers (medication decision-making) to others’: Gave medicines little thought, deferred decisions to their doctor or companion, unaware deprescribing is an option. Perceived they lacked knowledge about their health or medications. Majority male, frail. Reported fair or poor self-rated health				
Variables	Relative risk ratio	95% CI	*P* value	Relative risk ratio	95% CI	*P* value
Gender						
Male	*Ref*	*Ref*	*Ref*	*Ref*	*Ref*	*Ref*
Female	0.78	0.66–0.91	**<0.01**	0.48	0.39–0.58	**<0.001**
Education						
High school diploma or less	*Ref*	*Ref*	*Ref*	*Ref*	*Ref*	*Ref*
Trade school/some college/associate’s degree	0.78	0.65–.94	**0.01**	0.59	0.47–0.73	**<0.001**
Bachelor’s degree	0.58	0.47–0.71	**0.00**	0.41	0.31–0.53	**<0.001**
Master’s degree or higher	0.76	0.59–0.98	**0.04**	0.43	0.31–0.61	**<0.001**
Marital status						
Partner/married	*Ref*	*Ref*	*Ref*	*Ref*	*Ref*	*Ref*
Not partnered/married	0.90	0.67–1.22	0.51	1.12	0.78–1.62	0.52
Living situation						
Alone	*Ref*	*Ref*	*Ref*	*Ref*	*Ref*	*Ref*
With someone	0.87	0.64–1.20	0.40	1.12	0.76–1.64	0.57
Nursing home or retirement village	0.78	0.24–2.55	0.68	0.97	0.25–3.80	0.96
Health literacy						
None	*Ref*	*Ref*	*Ref*	*Ref*	*Ref*	*Ref*
A little bit	0.52	0.24–1.13	0.10	0.90	0.39–2.09	0.81
Somewhat	0.49	0.25–0.97	**0.04**	0.55	0.26–1.17	0.12
Quite a bit	0.39	0.21–0.75	**<0.01**	0.41	0.20–0.84	**0.02**
Extremely	0.35	0.18–0.66	**<0.01**	0.28	0.13–0.57	**<0.01**
Support for managing medications						
None	*Ref*	*Ref*	*Ref*	*Ref*	*Ref*	*Ref*
Occasional support	0.99	0.75–1.32	0.95	1.06	0.75–1.50	0.73
Complete assistance	1.45	0.95–2.20	0.09	1.32	0.80–2.18	0.28
Self-reported health						
Poor	*Ref*	*Ref*	*Ref*	*Ref*	*Ref*	*Ref*
Fair	1.25	0.86–1.82	0.24	1.04	0.65–1.68	0.86
Good	1.08	0.74–1.56	0.70	1.37	0.86–2.18	0.18
Very good	1.12	0.75–1.67	0.58	1.68	1.03–2.76	**0.04**
Excellent	1.13	0.66–1.95	0.66	3.04	1.64–5.63	**<0.001**
Previous experience with deprescribing (vs. none)	0.73	0.57–0.93	**0.01**	0.73	0.54–0.98	**0.03**
Age (years) (per 10-year increase)	1.26	1.08–s1.46	**<0.01**	1.47	1.23–1.77	**<0.001**
# of prescribed medications	1.00	0.99–1.00	0.31	0.99	0.98–1.00	0.13
# of over-the-counter medications/supplements	0.99	0.97–1.01	0.52	0.95	0.92–0.99	**0.01**
Medical Maximizing-Minimizing Preferences^b^ *(range: 1*–*7)*	1.05	0.99–1.12	0.10	0.88	0.82–0.95	**<0.01**
Beliefs about Medicines Questionnaire General^c^ *(α* = *0.85, range: 1*–*5)*	0.47	0.42–.53	**<0.001**	1.13	1.00–1.29	0.06
Agreement with hypothetical deprescribing recommendation *(range: 1*–*6)*	1.06	0.99–1.13	0.10	1.09	1.01–1.19	**0.03**
Polypharmacy attitudes^d^ *(range: 1*–*10)*	1.12	1.08–1.16	**<0.001**	0.95	0.90–1.00	**0.05**
Perceived harmfulness of deprescribing *(range: 1*–*10)*	1.04	1.00–1.08	**0.05**	1.00	0.96–1.05	0.87
Need for Certainty scale *(α* = *0.85, range: 1*–*5)*	1.11	1.01–1.23	**0.04**	0.96	0.86–1.08	0.54
Health Promotion scale^e^ *(α* = *0.87, range: 1*–*7)*	1.03	0.96–1.11	0.37	0.80	0.73–0.87	**<0.001**

a The qualitative typology descriptions are based on the previous qualitative research in which these typologies were created.^8^ The analyses were adjusted for the cluster effect by country.

b Higher values indicating a stronger preference towards medical interventions.

c Higher values indicating a stronger belief that medicines are over-used or harmful.

d Higher values indicating more positive attitudes.

e Higher values indicating a stronger preference for engaging in actions to promote health.

## Data Availability

The data that support the findings of this study are available on request from the corresponding author. The data are not publicly available due to privacy or ethical restrictions. The quantitative measure of the Patient Typology will be made available upon request.

## BOX 1 Summary of the significant results from the multinomial logistic regression

**Table:**

Categories	Selection of Typology 1 ‘Attached to medicines’ over Typology 2 ‘Would consider deprescribing’	Selection of Typology 3 ‘defers (medication decision-making) to others’ over Typology 2 ‘Would consider deprescribing’
Deprescribing	↓ previous experience with deprescribing ↑ perceived deprescribing as harmful	↓ previous experience with deprescribing ↑ slightly higher agreement with deprescribing recommendation
Attitudes towards medicines	↑ positive attitudes towards polypharmacy ↓ belief that medicines are over-used or harmful ↑ need for certainty	None
Knowledge about medicines and health	↓ confidence filling out medical forms ↓ obtained higher education levels	↓ confidence filling out medical forms ↓ obtained higher education levels ↓ desire to engage in actions to promote good health
Decision-making preferences	None	Leaning towards waiting and seeing
Characteristics	↓ female ↑ older age	↓ female ↑ older age
