# Supplementary material for: The Patient Typology about deprescribing and medication-related decisions: A quantitative exploration
Source: Basic Clin Pharmacol Toxicol. Author manuscript; Available in PMC 2024 Jun 20. (PMC11187678; doi:10.1111/bcpt.13911)
Supplement: Supplementary Tables [file NIHMS1988269-supplement-Supplementary_Tables.docx]

| **Supplementary Table 1. Baseline characteristics of participants who completed the survey and who responded to the Typology question** | | |
| --- | --- | --- |
|  | **Participants: complete survey**  **(n=5,311)** | **Participants: Typology question**  **(n=4,688)** |
|  | **n (%)** | **n (%)** |
| **Country** | | |
| Australia | 1,221 (23.0) | 1098 (23.4) |
| Netherlands | 1,250 (23.5) | 1021 (21.8) |
| United Kingdom | 1,340 (25.2) | 1231 (26.3) |
| United States | 1,500 (28.2) | 1338 (28.5) |
| **Gender** |  |  |
| Female | 2,506 (47.2) | 2226 (47.5) |
| Male | 2,792 52.6) | 2450 (52.3) |
| Missing | 13 (0.24) | 12 (0.3) |
| **Education status** | | |
| High school diploma or less | 1,619 (30.5) | 1425 (30.4) |
| Trade school/some college/Associate's degree | 1,843 (34.7) | 1671 (35.6) |
| Bachelor's degree | 1,155 (21.8) | 1057 (22.6) |
| Master's degree or higher | 574 (10.8) | 532 (11.4) |
| Missing | 120 (2.2) | 3 (0.1) |
| **Marital status** | | |
| Partnered/married | 3,409 (64.2) | 3073 (65.6) |
| Not partnered/married | 1,783 (33.6) | 1613 (34.4) |
| Missing | 119 (2.2) | 2 (0.5) |
| **Living situation** | | |
| Alone | 1,434 (27.0) | 1289 (27.5) |
| With someone | 3,558 (67.0) | 3221 (68.7) |
| Nursing home or retirement village | 22 (0.4) | 21 (0.5) |
| Missing | 297 (5.6) | 157 (3.4) |
| **Health literacy** | | |
| None | 96 (1.8) | 82 (1.8) |
| A little bit | 164 (3.1) | 145 (3.1) |
| Somewhat (potential for lower health literacy) | 502 (9.5) | 453 (9.7) |
| Quite a bit | 2,083 (39.2) | 1846 (39.4) |
| Extremely | 2,346 (44.2) | 2160 (46.1) |
| Missing | 120 (2.3) | 2 (0) |
| **Support for managing their medications** | | |
| None | 4,495 (84.6) | 4080 (87.0) |
| Occasional support | 410 (7.7) | 373 (8.0) |
| Complete assistance | 192 (3.6) | 169 (3.6) |
| Missing | 214 (4.0) | 66 (1.4) |
| **Self-reported health** | | |
| Poor | 238 (4.5) | 220 (4.7) |
| Fair | 1,325 (25.0) | 1209 (25.8) |
| Good | 2,284 (43.0) | 2041 (43.5) |
| Very good | 1,118 (21.1) | 1023 (21.8) |
| Excellent | 229 (4.3) | 195 (4.2) |
| Missing | 117 (2.2) | 195 (4.16) |
| **Previous experience with deprescribing (vs. none)** | 593 (11.2) | 538 (11) |
| Missing | 123 (2.3) | 3 (0.1) |
|  | Mean (SD) | Mean (SD) |
| **Age** | 71 (4.9) | 71 (4.9) |
|  |  |  |
| **# of prescribed medications** | 4.9 (8.7) | 4.9 (8.7) |
|  |  |  |
| **# of over-the-counter medications/supplements** | 2.1 (4.4) | 2.1 (4.3) |
|  |  |  |
| **Medical Maximizing-Minimizing Preferences^a^** *(Range: 1-6)* | 3.4 (1.4) | 3.4 (1.4) |
|  |  |  |
| **Beliefs about Medicines Questionnaire General^b^** *(α=0.85, Range: 1-5)* | 2.6 (0.8) | 2.6 (0.8) |
|  |  |  |
| **Agreement with hypothetical deprescribing recommendation** *(Range: 1-6)* | 4.8 (1.4) | 4.8 (1.4) |
|  |  |  |
| **Polypharmacy attitudes^c^** *(Range: 1-10)* | 4.0 (2.2) | 4.0 (2.2) |
|  |  |  |
| **Perceived harmfulness of deprescribing** *(Range: 1-10)* | 4.0 (2.4) | 4.0 (2.4) |
|  |  |  |
| **Need for Certainty scale** *(α=0.85, Range: 1-5)* | 3.6 (0.8) | 3.6 (0.8) |
|  |  |  |
| **Health Promotion** **scale^d^** *(α=0.87, Range: 1-7)* | 5.1 (1.1) | 5.1 (1.1) |

*Notes:* ^a^Higher values indicating a stronger preference towards medical interventions. ^b^Higher values indicating a stronger belief that medicines are over-used or harmful. ^c^Higher values indicating more positive attitudes. ^d^Higher values indicating a stronger preference for engaging in actions to promote health.

| **Supplementary Table 2: Results from the multinomial logistic regression analysis consistent with (or not) the qualitative typology hypotheses** | | |
| --- | --- | --- |
| **Categories and Measures** | **Consistency with (or not) the qualitative hypotheses** | |
| Measures | Selection of **Typology 1 *‘Attached to medicines’*** over Typology 2 *‘Would consider deprescribing’* | Selection of **Typology 3 *‘Defers (medication decision-making) to others’*** over Typology 2 *‘Would consider deprescribing’* |
| **Deprescribing** |  |  |
| 1. Agreement with deprescribing recommendation | Not significant | ↑ Slightly higher agreement with deprescribing recommendation  Consistent with the hypothesis |
| 2. Perception of harmfulness of deprescribing | ↑ Perceived deprescribing as harmful  Consistent with the hypothesis | Not significant |
| 3. Previous experience of deprescribing | ↓ Previous experience of deprescribing  Consistent with the hypothesis | ↓ Previous experience of deprescribing  Consistent with the hypothesis |
| **Attitudes towards medicines** | | |
| Measures |  |  |
| 4. Attitudes towards polypharmacy | ↑ Positive attitudes towards polypharmacy  Consistent with the hypothesis | Not significant |
| 5. Beliefs about medicines | ↓ Belief that medicines are over-used or harmful  Consistent with the hypothesis | Not significant |
| **Knowledge about medicines and health** | | |
| 6. Health literacy | ↓ Confidence filling out medical forms  Consistent with the hypothesis | ↓ Confidence filling out medical forms  Consistent with the hypothesis |
| 7. Education level | ↓ Obtained education levels  Consistent with the hypothesis | ↓ Obtained education levels  Consistent with the hypothesis |
| 8. Health promotion | Not significant | ↓ Desire to engage in actions to promote good health  Consistent with the hypothesis |
| **Decision-making preferences** | | |
| 9. Need for certainty | ↑ Need for certainty  Consistent with the hypothesis | Not significant |
| 10. Preferences for seeking medical care | Not significant | Leaning towards waiting and seeing  Consistent with the hypothesis |
| **Characteristics** | | |
| 11. Gender | ↓ Female  Consistent with the hypothesis | ↓ Female  Consistent with the hypothesis |
| 12. Age | ↑ Older age  Consistent with the hypothesis | ↑ Older age  Consistent with the hypothesis |
