# Supplementary material for: The Patient Typology about deprescribing and medication-related decisions: A quantitative exploration
Source: Basic Clin Pharmacol Toxicol. Author manuscript; Available in PMC 2024 Jun 20. (PMC11187678; doi:10.1111/bcpt.13911)
Supplement: Study Data [file NIHMS1988269-supplement-Study_Data.docx]

**Supplementary material: Survey questions included in the study**

| **Primary outcome: Quantitative measure of the Patient Typology** | |
| --- | --- |
| **Three typologies of older adults^1^** | **Description used in the current study**  Please read the following three descriptions. Then, select the description that is most like you. |
| *Typology 1: Attached to medicines*  Positive attitudes towards medicines, left decisions to their doctor, resistant to deprescribing. | - *My medicines are important, they keep me alive and help me to live well* - *I would not want to stop taking any of my medicines* - *My doctor and I talk about my medicines together* - *I trust my doctor to make decisions about my medicines* |
| *Typology 2: Would consider deprescribing*  Ambivalent attitudes towards medicines, preferred a proactive role in decision-making, were open to deprescribing. | - *I value what my medicines do for me* - *I wish I did not have to take as many medicines, and would stop taking one or more if I could* - *I know about my medicines – I ask my doctor or read the information leaflet or search online* - *I make decisions about the medicines I take or share the decision with my doctor* |
| *Typology 3: Defers (medication decision-making) to others*  Gave medicines little thought, deferred decisions to their doctor or companion, unaware deprescribing is an option. | - *I don’t really care much about my medicines, I take them as my doctor tells me to* - *My medicines are doing what they are supposed to do* - *If my doctor said I could stop a medication, I think that would be okay* - *Other people (e.g., my doctor or companion) make decisions about medicines for me* - *I don’t know much about my medicines* |

**Reference:**

1. Weir K, Nickel B, Naganathan V, et al. Decision-making preferences and deprescribing: perspectives of older adults and companions about their medicines. *The Journals of Gerontology: Series B*. 2018;73(7):e98-e107.

| **Variables** | **Items** |
| --- | --- |
| **Attitudes towards medications and deprescribing** | |
| Agreement with a hypothetical deprescribing recommendation from a primary care provider^2^ | *I think that Mrs. EF should follow her PCPs recommendation and stop taking [lansoprazole/simvastatin].*  1= Strongly disagree and 6= Strongly agree |
| Perceived potential harm of deprescribing^3,4^ | *I think that Mrs. EF stopping [lansoprazole/simvastatin] would be:*  1 = Not harmful and 10 = Very harmful |
| Beliefs about Medicines Questionnaire (BMQ)^5,6^: Beliefs about medicines general questions  NOTE: The BMQ has two sections: General (Harm and Overuse) and Specific (Necessity and Concerns). BMQ-Specific section was excluded due to high collinearity. Mean value was calculated across the 8 questions. | 1. *Doctors use too many medicines.* 2. *People who take medicines should stop their treatment for a while every now and again.* 3. *Most medicines are addictive.* 4. *Natural remedies are safer than medicines.* 5. *Medicines do more harm than good.* 6. *All medicines are poisons.* 7. *Doctors place too much trust on medicines.* 8. *If doctors had more time with patients they would prescribe fewer medicines.*   1= Strongly disagree and 5= Strongly agree |
| Attitudes towards polypharmacy (Ferrer et al., 2016)^7^ | *Mrs. EF takes 11 medicines. How positive or negative do you feel towards the number of medicines that Mrs. EF takes?*  1 = Very negative and 10 = Very positive |
| **Personality traits and health preferences** | |
| Medical Maximizer-Minimizer (MM1): Preferences for seeking more or less medical care (Scherer & Zikmund-Fisher, 2020)^8^ | *1 = I strongly lean towards waiting and seeing and 6 = I strongly lean towards taking action.* |
| Need for Certainty scale: Comfort or discomfort with uncertainty (Braithwaite et al., 2002)^9^  Note: Mean value was calculated across the 8 questions. | *1. I would rather have a medical test, and be certain about my future health, even if the result is bad news.*  *2. I would like to know now if I am likely to get ill so I can get used to the news.*  *3. If I didn’t have a medical test I would always be wondering whether I was going to develop a disease.*  *4. The relief I would get from a good result makes it worth the risk that the result is bad.*  *5. I think it is tempting fate to ask questions about future illness.*  *6. I would rather live with uncertainty than find out I was going to develop a disease.*  *7. Knowing the result of a medical test would mean I felt more in control.*  *8. It is better to know that I will develop a disease, even if I can’t prevent it.*  1= Strongly disagree and 5= Strongly agree |
| Health Promotion scale: Preference for engaging in actions to promote health, subscale of the Health Regulatory Focus Scale (HRFS) (Ferrer et al., 2017)^10^  NOTE: The HRFS has two subscales: Health Promotion and Health Prevention. The Health Prevention subscale was excluded due to high collinearity. Mean value was calculated across the 6 questions. | *1. I frequently imagine how I can achieve a state of “ideal health.”*  *2. I think of good health as a key to a happy life.*  *3. Doing healthy things gives me a sense of accomplishment.*  *4. When I engage in healthy behaviors, I am pleased with myself.*  *5. I would do anything to maintain a good, healthy body.*  *6. I admire people who do things that make them very healthy. 7. Knowing the result of a medical test would mean I felt more in control.*  1= Not at all and 7 = To a great extent |
| **Health characteristics** | |
| Self-rated general health^11^ | *In general, how would you rate your health today?*  1= Poor and 5= Excellent |
| Health literacy: Confidence filling out medical forms^12,13^ | *How confident do you feel filling out medical forms by yourself?*  1= Not at all and 5= Extremely |

**References:**

1. Weir K, Nickel B, Naganathan V, et al. Decision-making preferences and deprescribing: perspectives of older adults and companions about their medicines. *The Journals of Gerontology: Series B*. 2018;73(7):e98-e107.

2. Vordenberg SE, Weir KR, Jansen J, Todd A, Schoenborn N, Scherer AM. Harm and Medication-Type Impact Agreement with Hypothetical Deprescribing Recommendations: a Vignette-Based Experiment with Older Adults Across Four Countries. *Journal of general internal medicine*. 2022:1-10.

3. Dormandy E, Hankins M, Marteau TM. Attitudes and uptake of a screening test: The moderating role of ambivalence. *Psychology and Health*. 2006;21(4):499-511.

4. Scherer LD, Shaffer VA, Caverly T, et al. The role of the affect heuristic and cancer anxiety in responding to negative information about medical tests. *Psychology & health*. 2018;33(2):292-312.

5. Horne R, Weinman J. Patients' beliefs about prescribed medicines and their role in adherence to treatment in chronic physical illness. *Journal of psychosomatic research*. 1999;47(6):555-567.

6. Horne R, Weinman J, Hankins M. The beliefs about medicines questionnaire: the development and evaluation of a new method for assessing the cognitive representation of medication. *Psychology and health*. 1999;14(1):1-24.

7. Ferrer RA, Klein WM, Persoskie A, Avishai-Yitshak A, Sheeran P. The tripartite model of risk perception (TRIRISK): distinguishing deliberative, affective, and experiential components of perceived risk. *Annals of Behavioral Medicine*. 2016;50(5):653-663.

8. Scherer LD, Zikmund-Fisher BJ. Eliciting medical maximizing-minimizing preferences with a single question: development and validation of the MM1. *Medical Decision Making*. 2020;40(4):545-550.

9. Braithwaite D, Sutton S, Steggles N. Intention to participate in predictive genetic testing for hereditary cancer: the role of attitude toward uncertainty. *Psychology and Health*. 2002;17(6):761-772.

10. Ferrer RA, Lipkus IM, Cerully JL, McBride CM, Shepperd JA, Klein WM. Developing a scale to assess health regulatory focus. *Social Science & Medicine*. 2017;195:50-60.

11. DeSalvo KB, Bloser N, Reynolds K, He J, Muntner P. Mortality prediction with a single general self-rated health question. *Journal of general internal medicine*. 2006;21(3):267-275.

12. Chew LD, Bradley KA, Boyko EJ. Brief questions to identify patients with inadequate health literacy. 2004;

13. Wallace LS, Rogers ES, Roskos SE, Holiday DB, Weiss BD. Brief report: screening items to identify patients with limited health literacy skills. *Journal of general internal medicine*. 2006;21(8):874-877.
